# A Structured Low-Intensity Home-Based Walking Program to Improve Physical and Mental Functioning After Hospitalization for Severe COVID-19: A Pragmatic Nonrandomized Controlled Trial

**DOI:** 10.3390/jcm14196938

**Published:** 2025-09-30

**Authors:** Nicola Lamberti, Andrea Baroni, Giovanni Piva, Giulia Fregna, Nicola Schincaglia, Anna Crepaldi, Lorenzo Gamberini, Antonella Occhi, Sofia Straudi, Fabio Manfredini

**Affiliations:** 1 Department of Neuroscience and Rehabilitation, University of Ferrara, Via Luigi Borsari 46, 44121 Ferrara, Italy; nicola.lamberti@unife.it (N.L.); brnndr3@unife.it (A.B.); sofia.straudi@unife.it (S.S.); mdf@unife.it (F.M.); 2 Unit of Physical and Rehabilitation Medicine, University Hospital of Ferrara, 44124 Ferrara, Italy; giulia.fregna@unife.it (G.F.); nicola.schincaglia@edu.unife.it (N.S.); antonella.occhi@unife.it (A.O.); 3 Unit of Nephrology, University Hospital of Ferrara, 44124 Ferrara, Italy; annacrepaldi95@gmail.com; 4 PhD Program in Environmental Sustainability and Wellbeing, University of Ferrara, 44121 Ferrara, Italy; lorenzo.gamberini@unife.it; 5 Program of Vascular Rehabilitation and Exercise Medicine, University Hospital of Ferrara, 44124 Ferrara, Italy

**Keywords:** COVID-19, exercise therapy, low-intensity interval training, rehabilitation, walking, quality of life

## Abstract

**Background/Objectives**: We aimed to test whether home-based low-intensity interval training (LIIT) could be equally or more effective than traditional continuous walking advice (TWA) in a population hospitalized and healed from severe COVID-19. **Methods**: This pragmatic nonrandomized controlled trial (NCT04615390) enrolled patients admitted to intensive care units due to COVID-19 who at discharge from the hospital were given a choice between either a home-based LIIT program or TWA. The former received a structured LIIT walking (1:1 walk:rest ratio per 10 times) to be performed at a prescribed progressively increasing speed maintained with a metronome. The latter received TWA according to the guidelines (30 min or moderate intensity activity, 5 days/week). Outcome measures, collected at baseline, at the end of the 3-month training and at the 6-month follow-up, included 6 min walking distance (primary), lower limb strength, quality of life, depression and cognitive status. **Results**: From a total of 85 enrolled patients, 69 of them (LIIT *n* = 32; TWA *n* = 37) completed the study. Home exercise was safely executed with an 82% adherence for the LIIT group and 64% adherence for TWA. After the 3-month program, both groups significantly improved the 6MWD (LIIT: +87 m vs. TWA +42 m; *p* < 0.001) with a significant difference that was also maintained at follow-up (LIIT: +138 m vs. TWA +69 m; *p* < 0.001). No other significant between-group differences were noted. However, patients in the LIIT group significantly improved in the majority of the outcomes, while patients of TWA improved in only the primary outcome and the physical component of quality of life. **Conclusions**: Compared with TWA, LIIT walking was feasible, safe and associated with more favorable multidimensional recovery in COVID-19 survivors after hospitalization for severe pneumonitis.

## 1. Introduction

COVID-19, particularly in the most severe cases that require long-term bed rest and mechanical ventilation, leads to significant physical deconditioning, affecting the whole body [1,2,3,4]. Potential long-term complications were progressively recognized and reported, including enduring illness, cardiopulmonary disease, pain, fatigue, difficulty thinking, vertigo and insomnia, together with psychological sequelae [2,3,4].

It is currently known that the post-acute sequelae of SARS-CoV-2, also known as long COVID-19, affect a considerable number of individuals diagnosed with the infection. This condition can reveal itself with a wide range of symptoms (more than 100), more frequently including severe fatigue, orthostatic intolerance, shortness of breath, and reductions in exercise tolerance [5]. Even nonhospitalized patients who maintained exercise tolerance and cardiovascular function, levels of aerobic capacity and muscle strength lower than those in controls, accompanied by postural orthostatic tachycardia and myopathy, were invited to a cautious, even recommendable, exercise approach [6].

Several recommendations have been proposed for physical rehabilitation during the acute in-hospital phase, for returning to premorbid baseline physical activity and to return to sport activity for elite or leisure-time athletes [1,2,4,7]. However, in common clinical practice, at discharge from the hospital, patients who do not need a supervised intensive rehabilitation program are not routinely advised about what to do when they return home [8] or receive an exercise prescription to be carried out at home. Considering the positive impact of exercise in the management of several chronic diseases [9], tailored exercise training is considered an effective strategy to improve the symptoms and quality of life (QoL) for COVID-19 survivors after infection [10], recognizing the similarity of symptoms with the effects of cardiovascular deconditioning after bed rest, which leads to impaired maximal oxygen consumption and lower exercise capacity [5,11].

Several studies aimed at designing individualized, dynamic, cross-sectoral interventions in post-COVID-19 clinics with multiple specialties, including graded exercise, physical therapy, frequent medical evaluations, and cognitive behavioral therapy [2,4,12,13,14,15,16]. To this end, several meta-analyses already determined the positive impact of exercise in this population [16,17,18,19], even though a limited adherence and huge variability in the results were reported. Therefore, appropriate programs, whether supervised or home-based, are necessary when considering the well-known barriers to exercise [20] and also of the fears and concerns raised among the patients themselves about the tolerability and possible harms of exercise [10]

With this in mind, we hypothesized that a structured low-intensity interval (LIIT) walking program prescribed at the hospital and executed at home, previously successfully tested in diseases where pain, fatigue and deconditioning are common limiting symptoms [21,22,23,24,25,26,27], could be transferred to patients admitted to intensive or subintensive care units and healed from severe COVID-19. The aim of this nonrandomized trial was to test feasibility and safety of the program together with its effectiveness on exercise capacity and QoL with respect to traditional continuous walking advice (TWA), as recommended by scientific societies [5,28].

## 2. Materials and Methods

This prospective nonrandomized clinical trial (NCT04615390) was conducted at the Unit of Physical and Rehabilitation Medicine of Ferrara University Hospital between November 2020 and December 2022.

The Local Ethics Committee CE-AVEC approved the study (number 539/2020) on 20 May 2020. Written informed consent was obtained from all the participants. The study was conducted in accordance with the principles of the Declaration of Helsinki. The results of this trial are reported according to the CONSORT guidelines [29].

### 2.1. Subjects

Patients who recovered from COVID-19 infection and had required hospitalization in intensive or sub-intensive care units were encouraged to take part in the study. Other inclusion criteria were age greater than 18 years, male or female sex, capacity to walk for at least 20 m, not needing adjunctive oxygen therapy, and absence of clinical conditions contraindicating exercise training.

### 2.2. Interventions

Upon verification of eligibility, patients underwent baseline outcome-measure sessions, and were then asked to choose between two training programs: structured in-home low-intensity walking or traditional walking training according to American College of Sports Medicine (ACSM) guidelines. Both programs lasted for 3 months.

### 2.3. Structured Low-Intensity Interval Training

Patients selecting this option were provided with a detailed exercise prescription to be executed at home, in a corridor inside their house, or on a treadmill when available. The LIIT program encompassed two daily interval walking sessions at a prescribed speed. Patients were asked to walk at the arranged speed for one minute, interspersed with a 1 min resting period, to be repeated 10 times, either in the morning or in the afternoon/evening.

The training speed was converted into walking cadence, controlled by a metronome (in the form of a freeware digital application on a smartphone) and progressively increased from 60 to 99 steps/minute (+3 steps/min every week). During the first testing session, patients were instructed to walk in rhythm with the metronome [22,23]. Patients with a treadmill started training at the same corresponding speed as those in the metronome group (1.5 kmh^−1^), with a weekly increase of 0.2 kmh^−1^ per week until reaching the maximal value of 4.1 kmh^−1^. Each patient was given a training log in which to record the performance of the exercises and report any symptoms, to calculate adherence to the program.

### 2.4. Traditional Walking Advice

Patients who opted for this program received advice to walk outdoors or on a treadmill for at least 30 min, 5 days per week at a moderate intensity, according to the ACSM guidelines on exercise prescription [28]. Patients were therefore advised and encouraged to perform aerobic activities (in particular, walking) at a moderate level of effort (approximately 13–14 out of 20 on the Borg scale).

Each patient received a daily log to be returned during the subsequent visit, where they could report the actual program execution and any possible related symptoms (e.g., dyspnea, fatigue, etc.).

### 2.5. Outcome Measures

**Figure 1:**
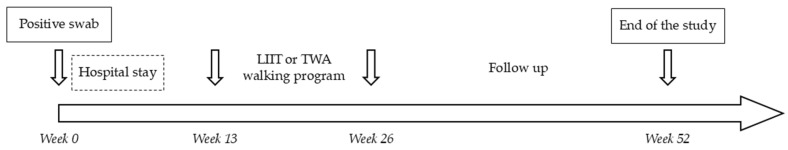
Timeline of the study.

The outcome measures were recorded at the time of entry (13 weeks after the first COVID-19-positive swab, T0), after 6 months (or 26 weeks, T1) and at the 52-week follow-up (T2) by an operator blinded to the patients’ allocation. The study timeline is reported in Figure 1.

The primary outcome measure selected for the study was the 6 min walk test, which was performed according to published standards [30]. Patients were instructed to walk on the same 21 m corridor as much as possible for 6 min, with the possibility of resting in cases where it was impossible to continue walking. The total distance covered (6 min walking distance, 6MWD) was noted. During the test, the heart rate (HR) and oxygen saturation (O2Sat) were continuously recorded by a pulse oximeter (Oxy-50, GIMA, Gessate, Italy). Finally, the minimal and maximal HRs and O2Sats were collected, as were their increases or decreases during the test (ΔHR and ΔSpO2).

The secondary outcomes included lower limb strength measured through the 30-s sit-to-stand test (30STS). With the patients sitting in a standard chair with their arms folded across the chest, they were asked to stand back as many times as possible in 30 s. The number of completed sit–stand–sit cycles was recorded.

The QoL was also assessed through the Italian version of the Medical Outcomes Study 12-Item Short-Form Health Survey (SF-12) [31]. The questionnaire includes 12 items concerning various physical and psychological aspects in the life of patients, which are ultimately summarized into physical component summary (PCS) and mental component summary (MCS) scores.

Aspects related to anxiety and depression were tested with the Beck Anxiety Inventory (BAI) and Patient Health Questionnaire-9 (PHQ-9). The BAI is a self-reported assessment of anxiety symptoms and has a total of 21 items, each of which is rated on a scale of 0–3. The total BAI score is obtained by summing the scores from all 21 questions [32]. The PHQ-9 is a self-report questionnaire used to screen for and measure the severity of depression. It consists of 9 items, and each item is scored from 0 to 3. The total PHQ-9 score is derived by summing the scores from all 9 questions [33].

Cognition was tested through the Montreal Cognitive Assessment (MoCA). This tool assesses various cognitive domains, including executive functions, visuospatial/constructional skills, naming, memory, attention, language, abstraction and orientation. The maximum score is 30, with a score below 26 suggesting potential cognitive impairment [34].

Finally, sleep quality was tested through the Pittsburgh Sleep Quality Index (PSQI). It comprises 7 sleep components, each of which is scored from 0 to 3, with a higher score indicating poorer sleep quality or greater difficulty [35].

### 2.6. Statistical Analysis

The data distribution was verified through the Shapiro–Wilk test. The data are then presented as the means ± standard deviations or medians (interquartile ranges) or mean (95% confidence interval) according to their parametric or nonparametric nature.

Baseline comparisons between the two groups of patients were carried out via independent samples *t* tests or Mann–Whitney tests for continuous variables or chi-square tests for categorical variables.

Within-group comparisons at the end of training programs were performed with paired-samples *t* tests or Wilcoxon tests accordingly. Between-group comparisons for variations in all outcomes were assessed with one-way analyses of variance or Kruskal–Wallis tests. The overtime analyses at the three time points were also tested with a two-way analysis of variance (factors: group, time).

Effects size were calculated using Cohen’s d for both within- and between-group analyses; the results were interpreted according to the published thresholds [36,37].

The data were analyzed via a per-protocol approach. We opted to not replace the missing data, which were not missing at random but were related to the unwillingness of the patients to undergo another hospital evaluation. In addition, a percentage of missing data of less than 20% ensures the maintenance of the external validity of the data.

A *p* value < 0.05 was considered significant. Statistical analysis was performed with MedCalc^®^ Statistical Software version 23.2.6 (MedCalc Software Ltd., Ostend, Belgium).

## 3. Results

**Figure 2:**
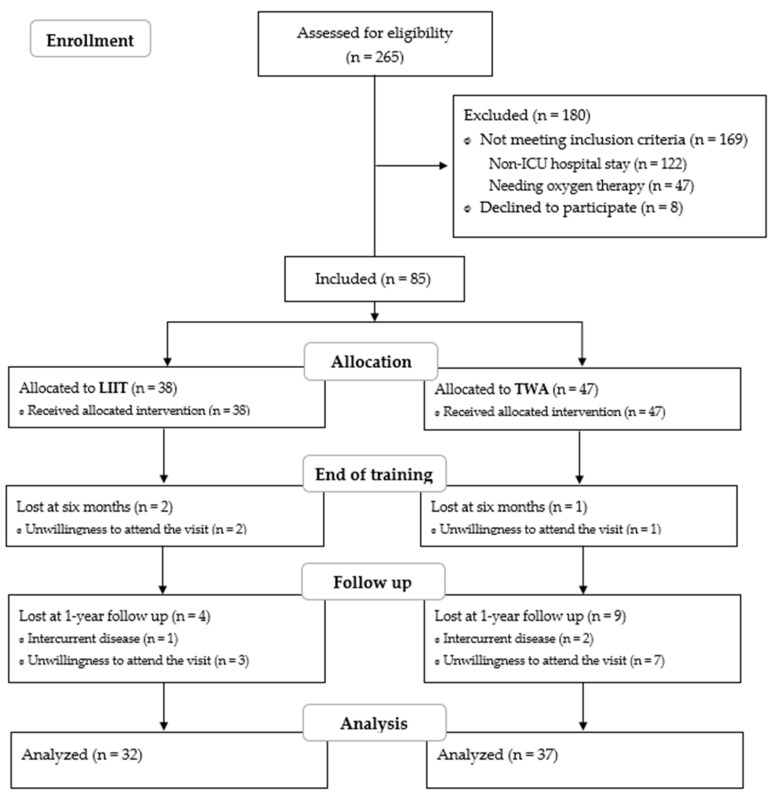
Study flow diagram.

Two hundred sixty-five patients were considered for inclusion in this study. One hundred eighty patients were excluded, and a final sample of eighty-five patients signed the informed consent form and agreed to participate in the trial. The reasons for exclusion are reported in Figure 2.

Among the included patients, 38 chose structured in-home walking training, whereas the remaining 47 selected the TWA option.

Three patients (two belonging to the LIIT group and one to the TWA group) did not attend the T1 follow-up visit, which was scheduled three months after enrollment. Moreover, 13 patients (4 in the LIIT group and 9 in the TWA group) did not show up at the 1-year follow-up visit (Figure 1). The main reason reported was the unwillingness to return to the hospital after a prolonged stay. These patients were recalled within the following four weeks to check if they had changed their decision; afterwards, they were thanked for their participation and excluded from the following analyses.

The final sample therefore included 69 patients, 32 from the LIIT group and the remaining 37 from the TWA group. This sample of patients did not present any baseline differences, except for lower MoCA scores in the LIIT group (Table 1).

### 3.1. Training Features

The whole population of the 69 patients analyzed did not report any significant discomfort or adverse reactions related to training execution. Fewer than 10% of patients, all belonging to the TWA group, reported light dyspnea during walking. Two patients reported fatigue after the walking sessions (one for each group).

Eighteen patients did not return the daily log; the certification of the training executed was therefore derived and estimated from a direct interview and, when possible, certified by a caregiver.

The LIIT group executed a median of 82% (range 65–100) for the walking sessions with respect to those prescribed, resulting in a median of 1476 min (range 1170–1800) for indoor walking per capita at a slow, progressively increasing speed.

The TWA group carried out a median of 64% (range 21–100) for the physical activity sessions, with a median of 1155 min walked (range 441–1800) per capita at moderate intensity, as recommended.

### 3.2. Outcomes at the End of Treatment (T1)

**Figure 3:**
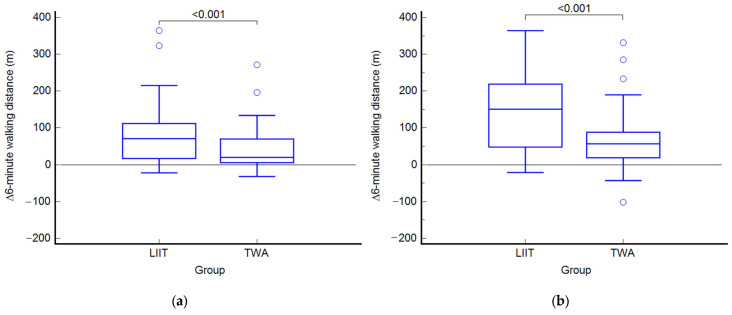
(**a**) Variations in the primary outcome at T1 respect to T0 in the two groups; (**b**) variations in the primary outcome at T2 respect to T0 (right panel) in the two groups.

After the 3-month program, both groups significantly improved the 6MWD (LIIT: +87 m vs. TWA +42 m), with LIIT achieving a 45 m improvement, significantly greater than TWA (*p* < 0.001) (Figure 3).

No other significant variations were observed for the other secondary outcomes, although the LIIT group reported better improvements in all the outcomes. In fact, a favorable significant variation was observed for the MoCA score in favor of the LIIT group; however, considering the baseline imbalances for this outcome between the two groups, when proper analysis of covariance was applied, the difference disappeared (*p* = 0.42).

### 3.3. Within-Group Variations at the End of Treatment (T1)

Patients enrolled in the LIIT obtained significant within-group variations from baseline to the end of the program for the primary outcome 6MWD (from 296 to 383 m; *p* < 0.001), ΔO2Sat (from −2.0 to −1.0%; *p* = 0.002), 30STS (from 8.2 to 11.2 repetitions; *p* < 0.001), PCS-12 (from 39.6 to 47.2 points; *p* < 0.001) and MoCA (from 22.4 to 23.6 points; *p* = 0.035).

Patients in the TWA group showed significant within-group improvements in the 6MWD (from 340 to 382 m; *p* = 0.001) and PCS-12 (from 42.5 to 46.9; *p* = 0.035).

The data are presented in Table 2 and Supplementary Materials.

### 3.4. Outcomes at Follow-Up (T2)

After the 6-month follow-up at the end of the training program, the between-group comparison highlighted a significant difference in the primary outcome in favor of the LIIT group (*p* < 0.001), which exhibited a 68 m greater improvement than did the TWA group.

No other between-group significant differences were observed, although more favorable improvements at follow-up were noted for all the secondary outcomes in favor of the LIIT group.

### 3.5. Within-Group Variations at Follow-Up (T2)

Patients enrolled in the LIIT maintained significant improvements with respect to baseline for the 6MWD (*p* < 0.001), ΔO2Sat (*p* = 0.012), 30STS (*p* < 0.001), PCS-12 (*p* = 0.022) and MoCA (*p* = 0.041); in addition, significant variations in sleep quality were observed (*p* = 0.031). Moreover, a significant difference with respect to T1 was noted for the 6MWD and 30STS.

Patients in the TWA group showed significant within-group improvements in the 6MWD (*p* < 0.001) and PCS-12 (*p* < 0.001), which were different at T2 than at baseline and T1 (Table 2 and Supplementary Materials).

### 3.6. Power Calculation

In the absence of a previously published study using a similar program, a post hoc power calculation was performed. For the primary outcome at the end of the exercise program, a combined power of 95.9% was obtained, considering the mean deviation from baseline to the end of treatment for the two groups.

## 4. Discussion

The present study, which was conducted in a population of adults hospitalized for severe COVID-19 infection, confirmed that a structured LIIT program executed at home was safe, feasible with good adherence and more effective at improving functional capacity than continuous walking advice, as recommended by the guidelines.

The scientific literature has called for targeted rehabilitation programs based on walking training for patients discharged from the hospital after contracting COVID-19 [2,3,7]. To address this topic, first, the patients’ response to exercise testing needs to be considered, since oxygen desaturation has been frequently reported in these patients during overground walking or chair raising, with a mean decrease in O_2_Sat values of approximately 3% [38,39,40], mirroring the values observed in our study. In addition, during walking, patients exhibited a significant increase in heart rate (mean value recorded: +12 beats per minute), although more than one out of three patients used β-blocker medications. Therefore, in patients hospitalized for COVID-19 pneumonitis, even two months after discharge from the hospital, an impaired response to walking that included a significantly lower distance walked (age-matched average value of 539 m [41]), a decrease in oxygen saturation and an increase in heart rate was reported [38,42]. Given these issues, exercise programs that physiologically address the exercise response of COVID-19 patients should be designed.

Various exercise programs have been proposed, mainly under supervision in the facility or at home by telecontrol. Indeed, various systematic reviews of trials have reported benefits of exercise-based rehabilitation in individuals who have COVID-19.

In particular, exercise-based rehabilitation interventions have been shown to be effective for long-term COVID-19-related symptoms characterized by dyspnea, fatigue, and depression, namely, mobility (mean improvement of the 6MWD: 95 m), respiratory function, quality of life and strength [18,19]. Another recent systematic review among adults (mean age 50 years) with long COVID-19 durations examined interventions lasting 2–8 weeks, mainly based on strength and aerobic exercise, and performed both under supervision and telerehabilitation or virtually confirmed the tolerability and benefit of exercise interventions [19]. Concerning older adults (aged 60 years or older), another meta-analysis comparing exercise-based interventions with usual/standard care reported significant yet smaller improvements in the 6MWD (mean difference +16 m) and 30STS (mean difference +4.1), as well as in fatigue, independence and mental health outcomes, particularly following exercise training, and in quality of life and pulmonary function in those who received respiratory rehabilitation [17].

In addition, recently published studies have focused on intervention models with home-based exercise under remote supervision. In a pragmatic randomized trial in adults discharged with post-acute COVID-19 conditions, a home-based online-supervised physical and mental health rehabilitation program improved QoL at three and twelve months compared with a group receiving best-practice usual care, which was based on a single online session of advice and support with a trained practitioner. This study revealed a high degree of adherence in approximately half of the enrolled population, and a total of 21 serious adverse events were reported (one possibly related to the experimental intervention) [43]. In individuals with residual disability post-acute COVID-19 and after inpatient rehabilitation, a 4-week home-based exercise program significantly improved effort tolerance only for people who had lower mobility at baseline [25]. Finally, another trial based on a remote 8-week exercise program reported improved mobility with respect to the group receiving usual care [44].

In the present study, we opted for a simple, well-established home-based exercise model based on an accurately prescribed but unsupervised graded walking. Graded exercise therapy has been proposed for various pathologic conditions associated with fatigue, with different outcomes [45,46]. We previously developed an effective graded LIIT walking program for patients with limited aerobic performance in terms of transport, delivery and exchange of oxygen [23,26,27,47], and these experiences have led us to adopt this model for COVID-19 patients affected by a mixed picture of deconditioning, bed rest and respiratory insufficiency. In our previous experiences with individuals with chronic conditions such as peripheral artery disease (PAD), end-stage kidney disease, and multiple sclerosis (MS), the LITT walking intervention participants performing below self-selected gait speed demonstrated significant improvements in exercise capacity [26], peripheral muscle oxygen utilization assessed via near-infrared spectroscopy [47,48], and mitochondrial function [49], alongside enhancements in mobility, respiratory mechanics, and reversal of muscle deconditioning in renal populations [49,50,51], and improved neuromuscular function in MS patients [52,53]; collectively, these findings support the utility of submaximal, low-fatigue [54] exercise strategies in rehabilitation settings for deconditioned populations [22,26]. The improvements in mobility and strength observed in the present study are in line with the values reported by us in those previous studies [21,26] and with values reported by other authors post-COVID-19, even though, considering the average age of our population and referring to the aforementioned review [26], the results are well above the average reported even compared with an active group and further improved at the 1-year follow-up.

In terms of effectiveness, one of the key features of the LIIT program is related to the progressive weekly increase in walking speed, according to the frequency, intensity, time and type (FITT) principles of training [28]. Indeed, the increase in the workload in terms of training velocity was well tolerated by the patients, who reported no adverse reactions or moderate-to-severe symptoms during training. This progressive load, empowered by the positive effects of interval training, mirrored the effects of glamorous high-intensity interval training in terms of enjoyment and time effectiveness [55]. Although a recent review concluded that interval training had the same effectiveness on functional capacity as conventional exercise training in sedentary young adults [55], training according to traditional walking advice, as recommended by the guidelines [28], is conducted at almost the same intensity throughout the entire training period. In our study, the majority of patients choosing TWA reported that they walked for a fixed time at moderate intensity covering the same distance each day, meaning that the training speed was always constant during the three months. Therefore, the observed difference between the two walking programs may be partly explained by the lack of training intensity progression in the TWA group, as recommended [28,56]. Another factor that may only partly explain the results obtained at the end of the programs is adherence to the training, which may have favored positive adaptations, owing also to the greater amount of total exercise volume. Indeed, patients choosing LIIT reported a mean adherence close to 85%, resulting in a median of 77 km (or 1476 min) walked inside home, whereas a median of 55% (62 km or 1155 min) of the TWA group did. This is not surprising, since very high compliance with a similar LIIT program in a diseased population has been reported [23,24,26,27], whereas low adherence (10 to 15%) to ACSM guidelines has been reported in older adults [57]. The absence of the typical barriers to exercise training of LIIT [58] has probably favored its execution since the program needed only a stop watch, a corridor and a chair to sit in during the resting phases. In addition, in-home execution allows patients to avoid the influence of weather, which impacts their participation in physical activity programs in adults [59]. Notably, in our study, approximately one out of two persons did not meet the minimum recommended physical activity levels [28], despite being provided with a training log to report the activity.

Another aspect worthy of discussion is the results obtained by the two groups at long-term follow-up, which was set one year after the first positive swab. Interestingly, the LIIT group maintained a significant difference for the majority of the outcomes with respect to baseline, also exhibiting a significant difference in the primary outcome between the groups. Indeed, recent reviews [60,61], despite confirming the short-term outcomes after exercise programs, failed to reach statistical significance in relation to the persistence of the benefits for all the outcomes analyzed, including mobility, strength and quality of life. Interestingly, a long-term legacy effect was observed in dialysis patients performing a similar exercise program for 6 months, and a higher persistence was noted in severe MS compared to traditional overground walking [53,54,55,56,57,58,59,60,61,62]. We do not know if patients in the LIIT group maintained a structured pattern of exercise at home after the end of the program; however, we believe that after 3 months of training, they acquired the tools and knowledge necessary to maintain an effective level of exercise at home.

This study has several limitations. First, it was designed and conducted as a nonrandomized study; this choice, despite slightly affecting external validity, allowed each patient to select the exercise program they preferred so that they were willing to perform continuously, favoring adherence. We also thought that the psychological condition post-COVID required freedom of individual choice. In addition, patients were recruited approximately one month after hospital discharge instead of immediately after discharge. Other concomitant physiotherapy treatments that may have had an impact on the results were allowed, despite only a minority of patients (<5%) participating in similar programs. In addition, the absence of a nonactive control group did not allow accounting for the physiological recovery that patients would have experienced anyway. Moreover, a portion of exercise diaries were not returned, making it more difficult to calculate precisely the minutes of exercise performed. Finally, a per-protocol analysis was conducted instead of an intention-to-treat approach to increase sensitivity.

## 5. Conclusions

In conclusion, if supervised interventions with continuous monitoring and tailoring ensure fidelity and patient safety [18], then low-cost, safe and simple protocols could meet another necessity, such as applicability to a broad population [18].

Compared with traditional walking advice, structured interval walking training was feasible, safe and associated with more favorable multidimensional recovery in COVID-19 survivors after hospitalization for severe pneumonitis. The low intensity of the program did not worsen the symptoms of fatigue in a population that usually experienced this symptom in everyday activities. The home-based structure of the program, which does not require a hospital stay, frequent visits or particular equipment, could be easily implemented at a low cost at each facility, as observed in other frail populations.

## Figures and Tables

**Table 1:** Baseline characteristics of patients in the two study groups.

	LIIT (*n* = 32)	TWA (*n* = 37)	*p*
Age, years	63 ± 11	65 ± 10	0.29
Males, *n* (%)	23 (72)	23 (62)	0.40
Obesity, *n* (%)	7 (22)	8 (22)	0.98
Hypertension, *n* (%)	15 (47)	24 (65)	0.14
Diabetes, *n* (%)	5 (16)	7 (19)	0.72
Total hospital stay, days	55 ± 35	52 ± 46	0.76
ICU stay, days	20 ± 17	19 ± 25	0.98
Mechanical invasive ventilation, *n* (%)	11 (34)	10 (27)	0.76
Noninvasive ventilation, *n* (%)	21 (66)	27 (73)	0.47
6MWD (m)	296 ± 101	340 ± 116	0.10
ΔHR (bpm)	11 ± 12	9 ± 11	0.19
ΔO_2_Sat (%)	−2 ± 3	−2 ± 2	0.66
30STS (reps)	8.2 ± 4.2	9.2 ± 4.2	0.34
PCS-12	39.6 ± 8.9	42.5 ± 8.6	0.18
MCS-12	49.5 ± 9.0	49.2 ± 8.8	0.90
BAI	7.5 ± 4.6	9.4 ± 7.8	0.23
PHQ-9	5.3 ± 2.9	6.7 ± 5.2	0.17
PSQI	11.3 ± 6.1	12.4 ± 5.4	0.42
MoCA	22.4 ± 3.7	24.3 ± 2.5	0.014

Abbreviations: ICU, intensive care unit; 6-MWD, 6 min walking distance; HR, heart rate; O2Sat, oxygen saturation; 30STS, 30-s sit-to-stand; PCS, physical component summary score; MCS, mental component summary score, BAI, Beck Anxiety Inventory; PHQ-9, Patient Health Questionnaire-9; PSQI, Pittsburgh Sleep Quality Index; MoCA, Montreal Cognitive Assessment.

**Table 2:** Outcome measures at the two time points in each group.

	LIIT (*n* = 32)			TWA (*n* = 37)					
	Baseline	End	Follow up	Baseline	End	Follow up	Between-group ΔT1-T0 Cohen’s d η^2^	Between-group ΔT2-T0 Cohen’s d η^2^	Two-way ANOVA p value
6MWT (m)	296 (260 to 333)	383 * (346 to 420)	434 *† (394 to 473)	340 (302 to 379)	382 * (349 to 415)	409 *† (366 to 453)	45 ‡ (8 to 82) d = 0.60 η^2^ = 0.083	68 ‡ (21 to 115) d = 0.71 η^2^ = 0.112	0.001
ΔHR (bpm)	11 (7 to 15)	9 (6 to 12)	8 (6 to 11)	9 (5 to 14)	10 (8 to 13)	9 (6 to 12)	−3 (−8 to 2) d = 0.45 η^2^ = 0.048	−3 (−9 to 4) d = 0.48 η^2^ = 0.055	0.24
ΔO_2_Sat (%)	−2.0 (−3.0 to −1.0)	−1.0 * (−1.6 to −0.5)	0.4 * (−1.1 to 2.1)	−1.6 (−2.3 to −0.9)	−1.1 (−1.6 to −0.5)	−0.6 (−1.2 to 0.0)	0.5 (−2.0 to 1.0) d = 0.28 η^2^ = 0.019	1.0 (−0.2 to 2.2) d = 0.75 η^2^ = 0.123	0.14
30STS reps	8.2 (6.7 to 9.7)	11.2 * (9.7 to 12.6)	13.1 *† (11.2 to 15.0)	9.2 (7.8 to 10.5)	10.9 * (9.7 to 12.2)	12.3 *† (10.7 to 13.9)	1.2 (−0.3 to 2.7) d = 0.39 η^2^ = 0.037	1.8 (−0.3 to 3.9) d = 0.41 η^2^ = 0.040	0.087
PCS-12	39.6 (36.4 to 42.9)	47.2 * (44.2 to 50.2)	44.9 * (41.6 to 48.2)	42.5 (39.6 to 45.3)	46.9 * (44.3 to 49.5)	46.3 * (43.1 to 49.5)	3.1 (−0.8 to 7.0) d = 0.40 η^2^ = 0.039	1.4 (−3.3 to 6.2) d = 0.14 η^2^ = 0.005	0.32
MCS-12	49.5 (46.3 to 52.7)	49.4 (46.6 to 52.2)	51.5 (48.7 to 54.4)	49.2 (46.3 to 52.2)	48.9 (45.8 to 52.1)	50.7 (47.3 to 54.0)	0.2 (−4.1 to 4.5) d = 0.02 η^2^ = 0.000	0.6 (−4.9 to 6.2) d = 0.06 η^2^ = 0.001	0.97
BAI	7.5 (5.8 to 9.2)	7.8 (5.1 to 10.6)	6.1 (4.0 to 8.1)	9.4 (6.8 to 12.0)	6.9 (3.5 to 10.4)	6.8 (4.6 to 8.9)	−2.8 (−1.7 to 7.4) d = −0.29 η^2^ = 0.021	−1.2 (−2.2 to 4.6) d = −0.17 η^2^ = 0.007	0.36
PHQ-9	5.3 (4.2 to 6.3)	4.7 (3.4 to 6.0)	4.4 (3.2 to 5.7)	6.7 (5.0 to 8.4)	5.2 (3.4 to 7.0)	4.3 * (3.2 to 5.3)	−0.9 (−1.6 to 3.4) d = −0.17 η^2^ = 0.007	−1.6 (−0.5 to 3.7) d = −0.36 η^2^ = 0.031	0.38
PSQI	11.3 (9.1 to 13.4)	9.7 (7.6 to 11.8)	8.3 * (6.6 to 10.0)	12.4 (10.6 to 14.2)	9.9 (7.6 to 12.2)	10.5 (8.3 to 12.7)	−1.0 (−2.2 to 4.1) d = −0.13 η^2^ = 0.004	1.1 (−4.0 to 1.8) d = 0.18 η^2^ = 0.008	0.37
MoCA	22.4 (21.1 to 23.8)	23.6 * (22.5 to 24.7)	23.8 * (22.5 to 25.1)	24.3 (23.5 to 25.2)	24.0 (23.1 to 24.9)	24.7 (23.7 to 25.7)	1.5 ‡ (0.0 to 2.9) d = 0.53 η^2^ = 0.066	1.1 (−0.7 to 2.9) d = 0.29 η^2^ = 0.021	0.19

* within-group *p* value < 0.05 with respect to baseline; † within-group *p* value < 0.05 with respect to end. ‡ between-group *p* value < 0.05. The data are reported as the means (95% confidence intervals). Abbreviations: 6-MWD, 6 min walking distance; HR, heart rate; O2Sat, oxygen saturation; STS, sit-to-stand; PCS, physical component summary score; MCS, mental component summary score; BAI, Beck Anxiety Inventory; PHQ-9, Patient Health Questionnaire-9; PSQI, Pittsburgh Sleep Quality Index; MoCA, Montreal Cognitive Assessment.

## Data Availability

The data supporting the findings of this study are available from the corresponding author upon reasonable request. Due to the ongoing nature of further investigation, some datasets are currently unavailable for public access.

## Supplementary Materials

The following supporting information can be downloaded at: https://www.mdpi.com/article/10.3390/jcm14196938/s1, Table S1: Within-group effect size (Cohen’s d) for all the study outcomes at the two timepoints of the study.

## Abbreviations

The following abbreviations are used in this manuscript:

QoL	Quality of life
LIIT	Low-intensity interval training
TWA	Traditional walking advice
ACSM	American College of Sports Medicine
6MWD	6 min walking distance
HR	Heart rate
O2Sat	Oxygen saturation
30STS	30-s sit-to-stand test
SF-12	Medical Outcomes Study 12-Item Short-Form Health Survey
PCS	Physical component summary
MCS	Mental component summary
BAI	Beck Anxiety Inventory
PHQ-9	Patient Health Questionnaire-9
MoCA	Montreal Cognitive Assessment
PSQI	Pittsburgh Sleep Quality Index
PAD	Peripheral artery disease
MS	Multiple sclerosis
