# Supplementary material for: A Structured Low-Intensity Home-Based Walking Program to Improve Physical and Mental Functioning After Hospitalization for Severe COVID-19: A Pragmatic Nonrandomized Controlled Trial
Source: J Clin Med. 2025 Sep 30;14(19):6938. doi: 10.3390/jcm14196938 (PMC12524472; doi:10.3390/jcm14196938)
Supplement: Supplementary file 1 [file jcm-14-06938-s001.zip › jcm-3837552-supplementary.pdf]

Supplementary Table S1. Within-group effect size (Cohen's d) for all the study outcomes at the two timepoints of the study.

	***LIIT* (*n*=32)**		***TWA* (*n*=37)**	
	Week 26 to Week 13	*Week 52 to Week 13*	*Week 26 to Week 13*	*Week 52 to Week 13*
6MWT (m)	0.85	1.31	0.39	0.56
30STS reps	0.75	1.10	0.50	0.66
PCS-12	0.88	0.58	0.57	0.42
MCS-12	-0.01	0.24	0.03	0.16
BAI	0.00	0.36	0.22	0.28
PHQ-9	0.00	0.28	0.39	0.73
PSQI	0.17	0.54	0.33	0.16
MoCA	0.35	0.38	-0.11	0.14
